# Economic consequences of Japanese schools’ recovery certificate policy for seasonal influenza

**DOI:** 10.1186/s12889-019-6600-0

**Published:** 2019-03-08

**Authors:** Shinya Tsuzuki

**Affiliations:** 1 Sapporo Maternity Women’s Hospital, Kita 13 jo Nishi 4 cho-me, Kita-ku, Sapporo, Hokkaido Postal code: 001-0013 Japan; 2 Disease Control and Prevention Center, National Center for Global Health and Medicine, Tokyo, Japan; 3 Faculty of Medicine and Health Sciences, University of Antwerp, Antwerp, Belgium

**Keywords:** Influenza, Japan, Cost analysis, Healthcare system, Recovery certificate

## Abstract

**Background:**

Like other countries, Japan experiences a seasonal influenza epidemic every year. In order to return to school after a influenza-related absence, most Japanese students are required to submit a recovery certificate (*chiyu-shoumeisyo* in Japanese). The objective of this study was to estimate the economic consequences of this practice.

**Methods:**

A cost analysis was conducted to estimate the additional costs incurred by the issuance of recovery certificates from a restricted societal perspective. The estimated number of influenza patients under 15 years old from the 2013/14 season to the 2017/18 season, the proportion of working mothers were used to calculate the estimated total number of recovery certificates issued per year. The cost of return visits to physicians and the cost for issuing certificates were included in the direct costs. Productivity loss was estimated using the mean monthly salary of women and was included in indirect costs.

**Results:**

The recovery certificate policy imposed an additional cost of 0.94 million USD per one million population. One-way deterministic sensitivity analysis demonstrated that the additional cost of the recovery certificate policy amounted to between 0.55 and 2.27 million USD per one million population. Probabilistic sensitivity analysis showed similar results.

**Conclusions:**

The recovery certificate policy has a substantial negative economic impact on the Japanese healthcare system and society from a restricted societal perspective.

## Background

A large number of Japanese children suffer from symptomatic seasonal influenza infection every year [1]. It is desirable that influenza patients refrain from having contact with other people for several days in order to minimize the risk of secondary infection. Although the length of time that a symptomatic influenza patient can spread the virus varies from person to person [2–6], the Japanese government prohibits children from going to their school or nursery school until five days have passed from the onset day of influenza symptoms in accordance with the School Health and Safety Act [7]. In Japan, influenza is usually differentiated from influenza-like illness (ILI) with the use of rapid influenza diagnostic tests (RIDTs) [8]. Almost all patients who are suspected of having ILI are examined by RIDTs, especially during peak influenza season. If a child’s RIDT is positive and they are diagnosed as having influenza, they must then stay at home for five days. If fever and other symptoms persist, the duration of attendance restriction is also extended until two days have passed after the decline of their fever. In addition, most children must submit a recovery certificate (*chiyu-shoumeisyo* in Japanese) to their school or nursery school in order for them to return. The recovery certificate is a document issued by a physician declaring that the individual is no longer contagious. At present, most schools in Japan do not permit students to return without a certificate [9].

This recovery certificate system involves various challenges. First of all, the five-day policy does not have sufficient scientific justification. Although a recent study reported that the mean duration of seasonal influenza’s viral shedding was about five days [10], the duration of shedding was different for each strain and depended on the age of the patient [11]. Furthermore, the presence of a virus does not always mean that a patient is infectious [12]. Therefore, it is difficult to judge whether a five-day absence is appropriate or not. Additionally, the U.S. Centers for Disease Control and Prevention has concluded that the duration of viral shedding is not related to fever [6]. If this result can be applied to cases of influenza among students in Japan, two additional days of isolation after one’s fever declines might not be appropriate.

Next, an additional challenge is that the documentation process seems to impose a societal and economic burden on both physicians and caregivers. This type of document must be issued by a medical doctor in Japan. Consequently, parents or other caregivers of children who have just had influenza must make an extra trip to their physician—a visit that comes attached with a fee. Healthcare costs for children are paid for by many local governments in Japan [13], but nevertheless, this process creates an economic burden from a healthcare payer’s or a societal perspective because these costs are ultimately covered by the national health insurance system. In addition, it is necessary to take the indirect costs into consideration. Caregivers have to take their children to their physician once they have recovered from influenza. As a result, caregivers are forced to take time off from work. This leads to productivity loss that, while not severe at the individual level, becomes substantial at the national level.

In addition, the recovery certificate does not seem to have any practical effect on the prevention of secondary infection. Physicians judge the day of recovery from face-to-face history taking. All children who revisit their clinic or hospital for requesting a certificate have already recovered, but their physicians cannot know the exact time of recovery [14]. Consequently, the only thing physicians can do is to trust the caregiver’s assessment of the recovery time. After this history taking process, physicians examine their patients and issue the certificate. There is almost no difference in this process from a self-assessment, as numerous Japanese infectious disease specialists have already pointed out [9, 14–17].

Due to the increased burden causes for healthcare facilities, the Ministry of Health, Labour and Welfare does not recommend the recovery certificate system [18], yet many schools in Japan require their students to submit a certificate nevertheless. In this study, I aimed to estimate the economic consequences of this recovery certificate policy.

## Methods

Direct and indirect annual costs of recovery certificates per 1,000,000 population at the national level were estimated from a restricted societal perspective. Direct costs include physician revisit consultation and documentation fees. Indirect costs include productivity loss estimated by the number of days of leave caregivers take for return doctor’s visits. For simplicity, I assumed an exchange rate of 1 USD = 110 JPY, based on the average exchange rate in 2018. I used data from the National Institute of Infectious Diseases (number of influenza patients) [19], national statistics from the Ministry of Health, Labour and Welfare (demographic data and health insurance costs) [20, 21], the National Institute of Population and Social Security Research (demographic data) [22], and the National Tax Agency (mean monthly salary) [23]. All data sources are freely available online.

The total number of recovery certificates issued each year was estimated by taking the total number of symptomatic influenza patients under 15 years old [19] combined with the proportion of households with children under 6 years old in which both parents work [20]. This is an important estimate because each household’s demand for a nursery school depends on whether both parents work or not. If children under 6 years old do not attend nursery school or kindergarten, then a recovery certificate would not be required. Almost all schools in Japan, including elementary schools (elementary education starts at 6 years old in Japan), nursery schools, and kindergartens, require that students who caught influenza submit a recovery certificate. As a result, the total number of documents required can be estimated by the sum of: (1) the total number of influenza patients between 6 and 14 years old and (2) the number of influenza patients under 6 years old multiplied by the proportion of households in which both parents work. Since there are no national data about the number of patients per year according to age, I substituted 5-year age group data instead and then subdivided those data into one-year age groups, according to the age structure of the Japanese population [22]. Thus, I can describe the total number of certificates per year with the following Eq. (1):

1 $$ {N}_t=\sum \limits_{j=0}^6\left({N}_j\ast {R}_j\right)+\sum \limits_{k=7}^{14}\left({N}_k\right) $$

Where *N*_*t*_ represents the total number of recovery certificates, *N*_*j*_ and *N*_*k*_ represent the number of influenza patients among children *j* or *k* years old, and *R*_*j*_ is the proportion of households in which both parents work and their youngest child is *j* years old.

**Fig. 1:**
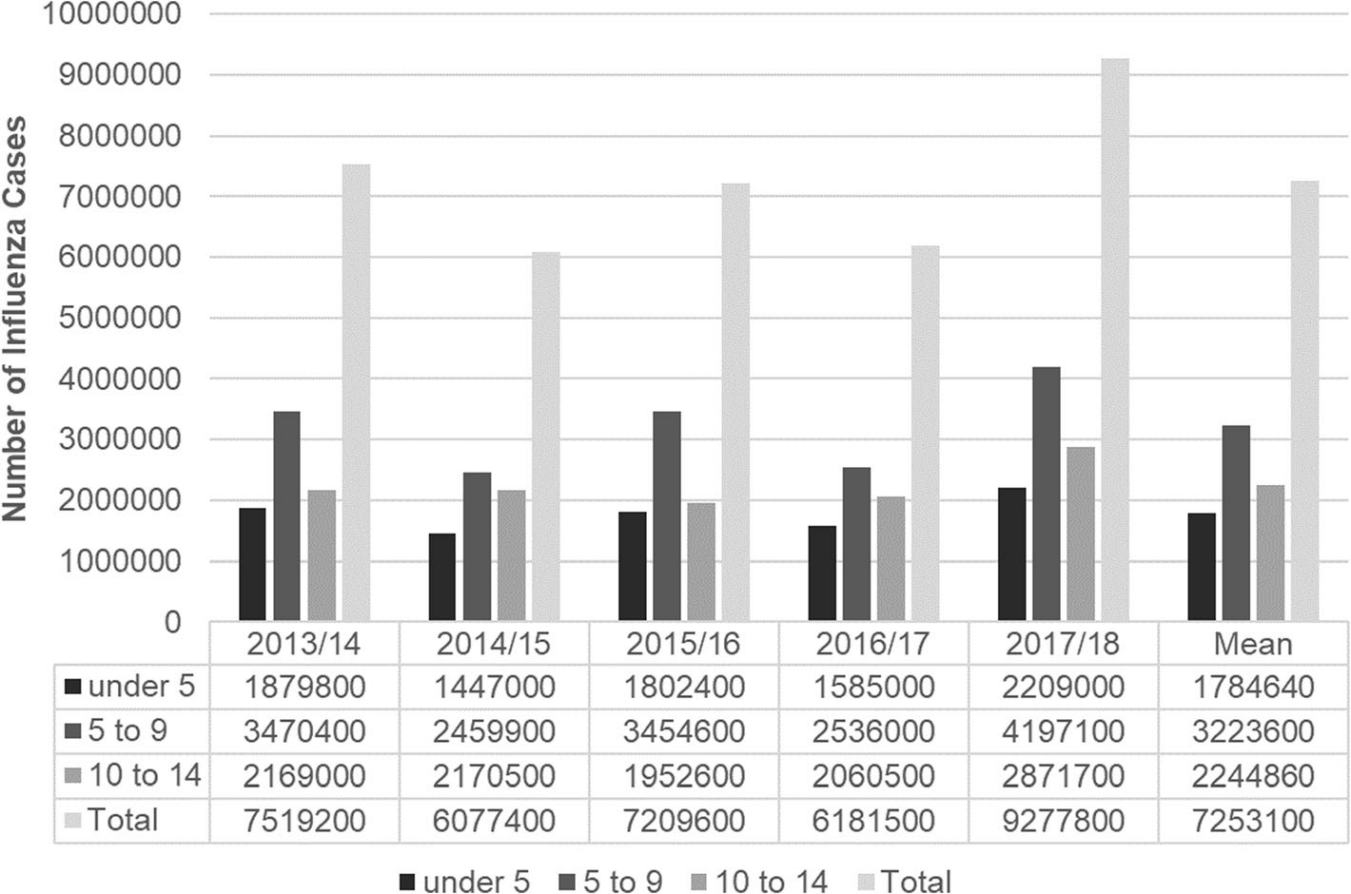
Estimated number of influenza patients under age 15

**Fig. 2:**
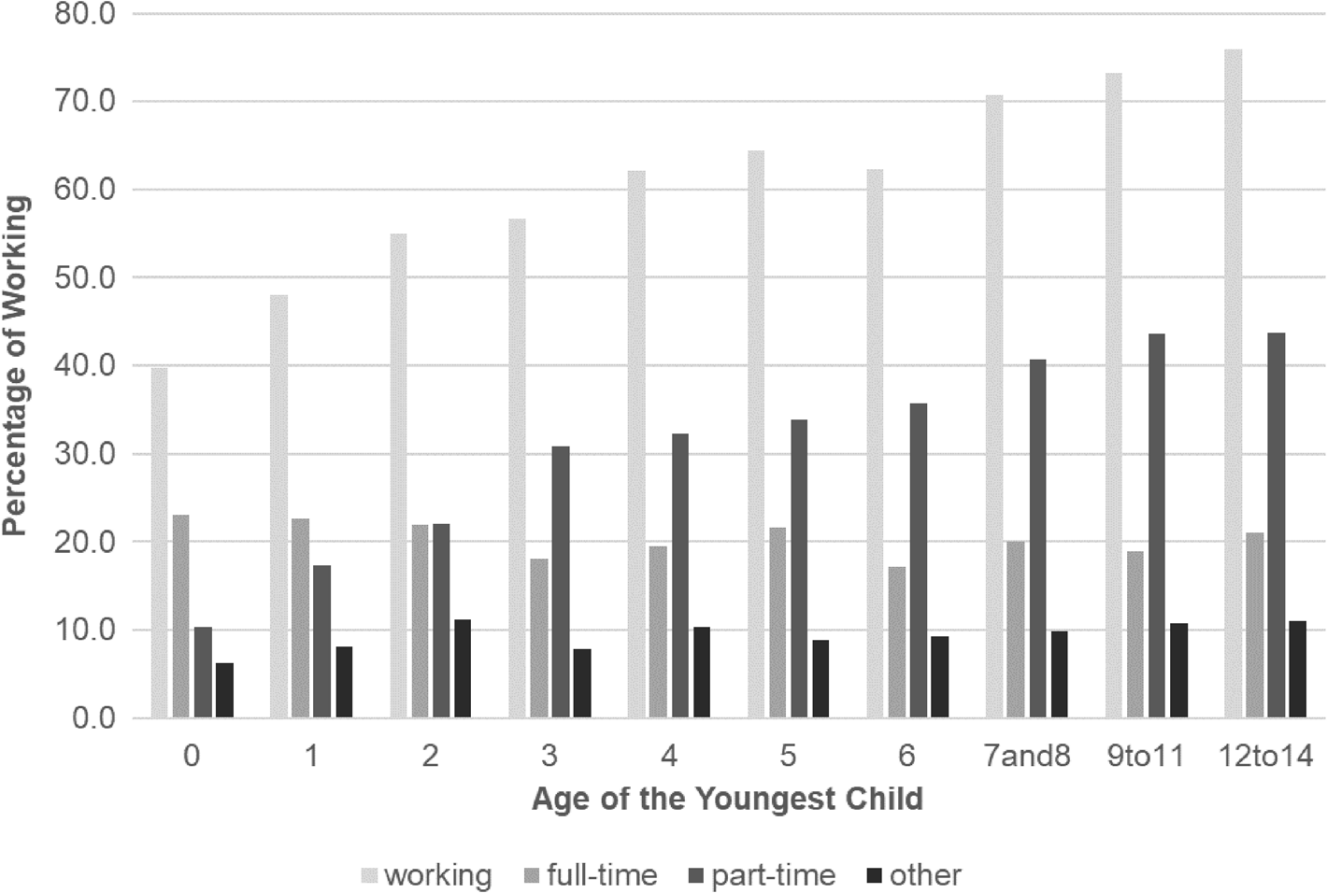
Proportion of working mothers classified by age of their youngest child

On average, Japan has about 7,253,100 influenza patients under 15 years old every year (Fig. 1). According to the data from national statistics, 54.3% of mothers whose youngest child was under 6 work, and 21.1% were full-time workers. On the whole, as their children get older, a larger proportion of mothers tend to work. Although the proportion of mothers who work full-time is constantly about 20%, the proportion of part-time workers increases as their children grow up (Fig. 2). In addition, 17.5% of households with children includes three or more generations. The average monthly salary of working women was 2187.8 USD.

In Japan, the physician fee for a revisit is 10.1 USD per person for patients under 6 years old and 6.6 USD per person for children 6 and older, both of which are set by the Japanese government [21]. In contrast, price setting for documentation fees is left to the discretion of each healthcare facility. As such, I have assumed a documentation fee of 5 USD, an assumption which is supported by the expert opinion of Board Certified Pediatricians of Japan Pediatric Society. Additionally, some physicians do not claim any documentation and/or consultation fee from patients. Therefore the total number of certificates and revisit consultations were adjusted by the proportion of physicians who do not claim such fees [24]. This proportion was determined using an information website for healthcare professionals in which over 200,000 Japanese physicians are registered to participate in surveys. Of those, 1277 physicians were randomly chosen by the website to participate in the survey and to answer a questionnaire about the recovery certificate. According to the survey results, 18% of physicians do not charge a fee to issue the certificate. In addition, 19% of physicians charge only a revisit consultation fee, while 63% charge both consultation fees and documentation fees.

Considering the survey results with Eq. (1), total direct costs of the recovery certificate policy can be described with the following Eq. (2):

2 $$ total\kern0.5em direct\kern0.5em cost={N}_t\ast \left({C}_c\ast {P}_c+{C}_d\ast {P}_d\right) $$

Where *C*_*c*_ and *C*_*d*_ represent the cost for revisit consultation fees and documentation fees, respectively, and *P*_*c*_ and *P*_*d*_ represent the proportion of physicians who claim revisit consultation fees and documentation fees, respectively. *N*_*t*_ represents the total number of certificates.

In order to calculate productivity loss, I used the mean monthly salary of working women from 2013 to 2018 [23]. Most parents request a recovery certificate in the morning to minimize the duration of both their own and their children’s absences from work and school, respectively. Then, after obtaining the document, they take their children to school, and go to work. Though it is difficult to estimate the exact duration of waiting time in outpatient clinics, the processes described above might be equivalent to a half-day of leave (from the beginning of work to lunch break). So, I estimated productivity loss corresponding to one half-day leave by taking half the amount of the mean monthly salary and dividing it by total working days.

Another important factor is how many caregivers must take off time from work to take their children to a physician. In the first place, stay-at-home parents and the unemployed can visit healthcare facilities without taking leave. Therefore, the total productivity loss caused by physician revisits can be represented as a product of the total number of certificates issued, the proportion of households in which both parents work, and a half-day of the mean daily salary.

Considering a caregiver’s employment status (full-time or part-time), parents who work full-time jobs always have to take time off to visit a physician. Some part-time workers might be able to visit a physician without taking leave, but others might have to take time off. My equation averages out these differences at the population level because I use mean salary which includes both full-time and part-time workers.

Additionally, it is important to note the role of grandparents. In Japan, retired grandparents play an important role in childrearing. Indeed, 72% of parents can request that their own parents take care of their children when they become sick [25]. In such cases, it is the grandparents who take their grandchildren to a healthcare facility, allowing the children’s parents to avoid taking time off from work. Also, I assumed that children aged 12 years and older (i.e. those who already have graduated from elementary school) can go to a healthcare facility by themselves, an assumption which is also supported by expert opinion of Board Certified Pediatricians of Japan Pediatric Society.

I did not include transportation fees in either the direct and indirect costs because there are a large number of private clinics in Japan and so people can usually visit a clinic near their home at little cost. In addition, a half-day of leave is assumed to be sufficient for obtaining a certificate because all patients are essentially healthy and require no further medical treatment or laboratory tests. I did not assume any discounting in my cost analysis because the outcomes are based only on costs themselves and analyses were conducted on the basis of a single-year assumption. Finally, I determined productivity loss caused by the recovery certificate policy by the equation:

3 $$ total\kern0.5em indirect\kern0.5em cost=\sum \limits_{j=0}^{12}\left({N}_j\ast {R}_j\right)\ast \left(1-{P}_{gp}\right)\ast W $$

Where *N*_*j*_ represents the number of influenza patients among children *j* years old and *R*_*j*_ is the proportion of households in which both parents work and their youngest child is *j* years old; *P*_*gp*_ represents the proportion of parents who can ask grandparents to take care of sick children; and *W* represents the lost productivity from taking a half-day of leave.

I conducted one-way deterministic sensitivity analyses for each of the five variables influential for the result (i.e. document cost, total number of documents issued, proportion of grandparents who can help parents, length of leave that parents have to take, and mean salary of working women). The lower and upper values of the total number of documents issued and the mean salary of working women were set as ±30% of the original assumption following a previous study [26] because there was no information about their range. As for the range of document costs, I set it between 0 USD to 50 USD because there are some healthcare facilities which offer free documentation for recovery certificates, while others charge a higher fee similar to other types of medical certificates [24]. I assumed the range for the length of leave parents have to take was between one hour and one day. I also conducted a probabilistic sensitivity analysis (PSA) including 1000 simulations with assumed distribution of parameter values (log-normal or triangular distribution). Values and distribution of each parameter are shown in Table 1.

**Table 1:** Values and distribution of each parameter

Variable	Value (unit)	Range	Distribution	Reference
Document fee	5 (USD)	0–50 (USD)	lognormal	[24]
Consultation fee for children under 6	10.1 (USD)	Fixed	NA	[21]
Consultation fee for children 6 or older	6.6 (USD)	Fixed	NA	[21]
Proportion of grandparents who can take care of sick children	0.72	0.504–0.936	triangular	[25]
Length of leave taken	0.5 (day)	0.125–1.0 (day)	triangular	Assumption
Mean monthly wage	2187.8 (USD)	1531.5-2844.2 (USD)	lognormal	[23]
Number of certificate issued^a^	5,937,692	4,156,384-7,719,000	triangular	[19, 20, 24]

^a^in the total population of Japan, per one season; *NA*: not applicable

All analyses were performed with R, version 3.5.1 (R Foundation for Statistical Computing, Vienna, Austria) [27].

## Results

Under the assumptions described in the previous section, the mean annual cost of the recovery certificate policy amounted to 939,872 USD per million population in Japan. Direct costs were estimated to be 430,737 USD and indirect costs were 509,135 USD per million population.

Deterministic, one-way sensitivity analyses demonstrated that the total additional costs ranged from 547,111 USD to 2,265,333 USD per million population (Table 2). Document fees played a comparatively important role in determining the total economic burden, but nevertheless, each variable did not change the results critically.

**Table 2:** Main result and results of deterministic one-way sensitivity analyses

	Direct cost	Indirect cost	Total cost
Original assumption	0.43	0.51	0.94
Document fees	1.76	0.51	2.27
	0.28	0.51	0.79
Number of document issued	0.56	0.66	1.22
	0.30	0.36	0.66
Proportion of grandparents who can take care of sick children	0.43	0.12	0.55
	0.43	0.90	1.33
Length of leave taken	0.43	1.02	1.45
	0.43	0.13	0.56
Mean monthly wage	0.43	0.66	1.09
	0.43	0.36	0.79

Unit: million USD per million population

Upper rows represent results of the upper parameter value, bottom rows represent results of the lower parameter value

**Fig. 3:**
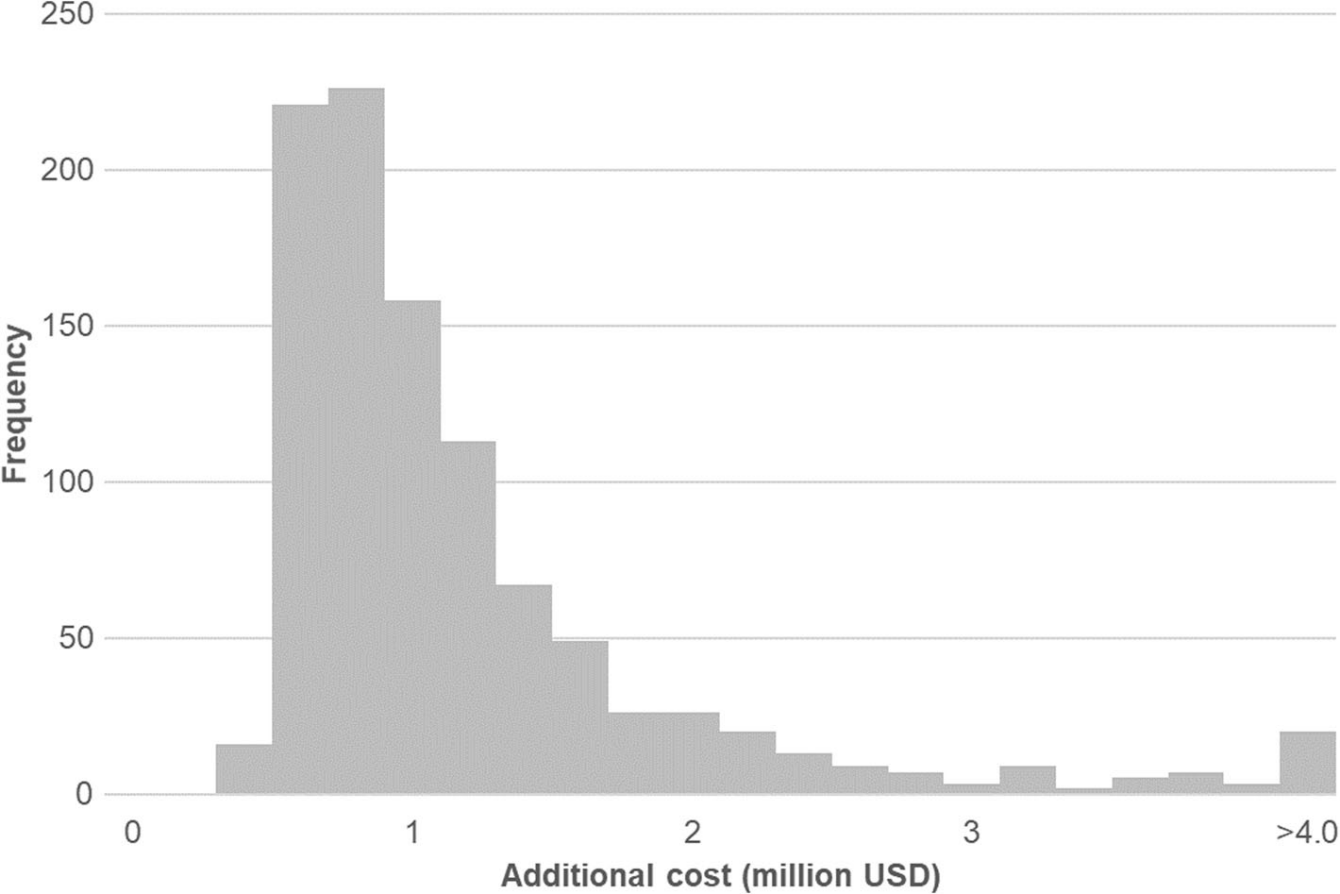
Results of probabilistic sensitivity analysis. A histogram of 1000 simulations of the probabilistic sensitivity analysis. Y axis represents the frequency of trials

Probabilistic sensitivity analysis demonstrated that the additional cost of the recovery certificate amounted to about million USD per million population in most cases (median: 847,193 USD, interquartile range: 610,724-1,235,824 USD). Figure 3 shows a histogram of 1000 simulation results.

## Discussion

To my knowledge, this is the first study to measure the economic burden imposed on Japanese society by the recovery certificate system. The results of this study should create an opportunity to consider the impact of this long-standing custom.

Currently, Japan is experiencing the effects of a rapidly aging society, and its national medical expenses are growing larger and larger. In fiscal year 2015, annual expenses for medical costs in Japan amounted to 3,268 billion USD per million population and this figure is expected to rise [28]. Although 0.94 million USD is quite small compared to total medical expenses, it is nevertheless a substantial burden on Japanese society and the benefit of the recovery certificate practice is uncertain.

As detailed in this paper, there is no justifiable reason for schools to demand that children submit a certificate to return after recovering from influenza. On the contrary, there is a possibility that these recovery certificates do more harm than good. I should note that the situation, as it is now, sees healthy parents taking their already recovered children to healthcare facilities when they need certificates. Needless to say, there are a large number of sick people at these facilities. These sick people include patients with infectious diseases, which exposes the healthy caregivers and recovered children to new risks of infection. If it were possible to estimate the risk of secondary infection caused by obtaining a certificate, the total negative economic impact would become larger still and the additional disease burden could be visualized. Both from the perspective of economics and clinical medicine, the recovery certificate policy appears to have a net negative impact on our society. In fact, the government of Okinawa Prefecture has already declared that it does not recommend using recovery certificates as a requirement for returning to school [29].

Additionally, most schools in Japan have also adopted a recovery certificate policy for other infectious diseases (e.g., varicella, mumps, and so forth). This type of response probably derives from the Japanese aversion to risk. From the standpoint of school administrators, outbreaks of any infectious disease in their own schools are to be completely avoided. However, their attitude makes it difficult to conduct an appropriate risk assessment. Presumably, school administrators do not know the actual impact of recovery certificates and believe they are beneficial. I believe appropriate and regular information updates from the government and healthcare professionals is needed to improve the current situation.

As with all other research, my study has some limitations. First, I conducted my analyses with incomplete data. For example, data about the number of influenza patients only contained 5-year age groups. As a result, there might be some discrepancy between the real number of schoolchildren who need a certificate and the estimated one. As 98% of 5 year old children belong to some kind of kindergarten or nursery school [30], the estimated total number of documents is expected to be similar to the actual one. Furthermore, only point estimates were available for some parameters, so I had to set range of parameter values arbitrarily. This might also impair the robustness of the results.

Second, our assumptions include the possibility of some under/overestimation. I did not include any transportation fees for revisits even though some caregivers must use public transportation or private cars. In addition, some parents request that their physicians prescribe drugs for the common cold during the same visit. Antitussives and expectorants are not expensive drugs, but it is likely that these drugs would never be prescribed if parents did not have to revisit their physician to obtain a certificate. As for overestimation, I assumed that all schools and nursery schools require children to submit a certificate. Although the exact number is not known, a small number of schools do allow children to return to school without a recovery certificate. Nevertheless, my assumptions (e.g., some physicians issue certificates without charging a fee) and the wide range of sensitivity analyses should compensate for such under/overestimates.

Third, our analysis neglected the possibility that the recovery certificate policy has any positive impacts for preventing secondary transmission of influenza. If the duration of the infectious period lasts longer than 5 days, isolation at home for more than 5 days might be beneficial for preventing the spread of infection. However, as I have already explained, physicians issue certificates based on information from patients’ caregivers. Consequently, patients could attend school on the same day if they did not need to submit any documents.

Considering these limitations, this analysis might sacrifice accuracy and robustness to some extent. Nevertheless, I believe that the results are reasonable, and worthy of note because quantitative analysis of the societal impact of the recovery certificate policy has been insufficient to date.

## Conclusion

As I have shown, the recovery certificate system, which is unique to Japan, has some negative economic and societal consequences. Reconsideration of this policy may enable us to reduce excessive primary healthcare costs.
